# Bedside measurement of changes in lung impedance to monitor alveolar ventilation in dependent and non-dependent parts by electrical impedance tomography during a positive end-expiratory pressure trial in mechanically ventilated intensive care unit patients

**DOI:** 10.1186/cc9036

**Published:** 2010-05-30

**Authors:** Ido G Bikker, Steffen Leonhardt, Dinis Reis Miranda, Jan Bakker, Diederik Gommers

**Affiliations:** 1 Department of Intensive Care Medicine, Erasmus MC, 's-Gravendijkwal 230, Rotterdam, 3015 GE, The Netherlands; 2 Helmholz-Institute for Biomedical Engineering, RWTH Aachen University, Pauwelsstraße 20, Aachen, D-52074, Germany

## Abstract

**Introduction:**

As it becomes clear that mechanical ventilation can exaggerate lung injury, individual titration of ventilator settings is of special interest. Electrical impedance tomography (EIT) has been proposed as a bedside, regional monitoring tool to guide these settings. In the present study we evaluate the use of ventilation distribution change maps (ΔfEIT maps) in intensive care unit (ICU) patients with or without lung disorders during a standardized decremental positive end-expiratory pressure (PEEP) trial.

**Methods:**

Functional EIT (fEIT) images and PaO_2_/FiO_2 _ratios were obtained at four PEEP levels (15 to 10 to 5 to 0 cm H_2_O) in 14 ICU patients with or without lung disorders. Patients were pressure-controlled ventilated with constant driving pressure. fEIT images made before each reduction in PEEP were subtracted from those recorded after each PEEP step to evaluate regional increase/decrease in tidal impedance in each EIT pixel (ΔfEIT maps).

**Results:**

The response of regional tidal impedance to PEEP showed a significant difference from 15 to 10 (*P *= 0.002) and from 10 to 5 (*P *= 0.001) between patients with and without lung disorders. Tidal impedance increased only in the non-dependent parts in patients without lung disorders after decreasing PEEP from 15 to 10 cm H_2_O, whereas it decreased at the other PEEP steps in both groups.

**Conclusions:**

During a decremental PEEP trial in ICU patients, EIT measurements performed just above the diaphragm clearly visualize improvement and loss of ventilation in dependent and non-dependent parts, at the bedside in the individual patient.

## Introduction

Mechanical ventilation is critical for the survival of most patients with respiratory failure admitted to the ICU, but it has become clear that it can exaggerate lung damage and may even be the primary factor in lung injury [1]. Protective ventilatory strategies to minimize this lung injury include reduction of tidal volume and prevention or minimization of lung collapse and overdistension by adequate setting of the positive end expiratory pressure (PEEP) [2]. Currently, PEEP setting is often guided by global lung parameters such as arterial oxygenation or global compliance, which are not specific for regional lung collapse or overdistension [3]. If a regional monitoring tool for lung collapse and overdistension would be available at the bedside, this would aid optimization of ventilator settings in individual patients.

Electrical impedance tomography (EIT) is a noninvasive, real-time imaging method that provides a cross-sectional ventilation image of the lung [4-6]. It is based on the measurement of lung tissue impedance by injection of small currents and voltage measurements, using electrodes on the skin surface. Recently, different studies described ventilation distribution change maps to evaluate lung collapse or overdistension [7-9]. Costa *et al*. described the use of these ventilation distribution change maps in two ICU patients [7]. They introduced an algorithm in which ventilation at a PEEP level is expressed as a percentage from maximal ventilation as seen after a lung recruitment maneuver. In lung-lavaged pigs, Meier *et al*. composed functional EIT images or ventilation distribution maps by subtracting the EIT images from two PEEP levels to show improvement or loss of regional ventilation between these two PEEP levels [9]. As the clinically set PEEP is often guided by decremental PEEP trials, it would be of interest to evaluate the ventilation distribution change maps during this procedure.

In the present study we evaluate the use of ventilation distribution change maps in ICU patients with two distinct types of lung conditions: with or without lung disorders during a standardized decremental PEEP trial. Furthermore we investigated if the EIT measurements at the bedside may visualize alveolar recruitment and derecruitment in the dependent and non-dependent lung regions.

## Materials and methods

Following approval by the local institutional human investigations committee, patients were enrolled after providing informed consent from their legal representatives. The study population consisted of 14 mechanically ventilated patients on a mixed ICU. In eight of these patients, end-expiratory lung volume was also measured and these data have recently been published [10]. For all 14 patients, chest x-rays and, if available, CT-scans were retrospectively evaluated and related to clinical history and data to divide these patients into two groups; without lung disorders (Group N) and with lung disorders (Group D). Patients were regarded to be without lung disorders when no clinical signs of respiratory failure, pneumonia or significant atelectasis were present. The group with lung disorders was defined as PaO_2_/FiO_2 _ratio <300 mmHg and proven pneumonia or abdominal sepsis. All patients were well sedated and ventilated in pressure-controlled mode, without spontaneous breathing activity. All patients were ventilated with constant driving pressures throughout the procedure, mean applied driving pressure was 12 cm H_2_O (range 9 to 18) in Group N and 16 cm H_2_O (range 12 to 21) in Group D. Exclusion criteria for participation in the study were: pneumothorax, severe airflow obstruction due to Chronic Obstructive Pulmonary Disease (COPD) (defined as forced expired volume in 1 s or vital capacity below predicted value minus 2 SD), lung transplantation, thoracic deformations and severe cardiovascular instability.

**Figure 1:**
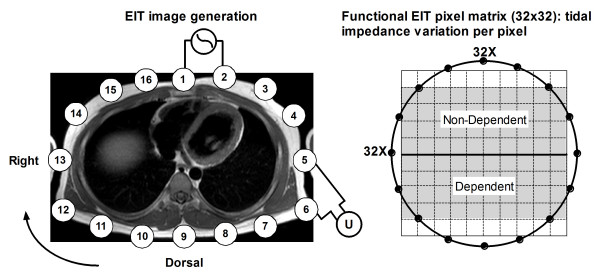
**Principle of electrical impedance tomography (EIT) and the functional EIT image (fEIT)**. Electrical excitation currents are applied between pairs of adjacent surface electrodes (1 to 16); the resulting voltages are measured between the other electrodes (U). In the fEIT image, impedance variation induced by the tidal volume is divided into a 32 × 32 matrix. Each pixel contains the individual tidal impedance variation, creating an image of ventilation distribution. The ventral to dorsal oriented ROIs are marked in gray in the right panel.

In all patients, impedance measurements were performed during two minutes with a silicone belt with 16 integrated electrocardiographic electrodes placed around the thoracic cage at the fifth or sixth intercostal space (Figure 1), connected with an EIT device (EIT evaluation kit 2, Dräger, Lübeck, Germany). EIT data were generated by application of a small alternating electrical current of 5 mA at 50 kHz. After baseline measurements at the clinical set ventilator settings (Table 1), PEEP was increased to 15 cm H_2_O. After a steady state of 15 minutes PEEP was decreased stepwise each 10 minutes to 10, then to 5 and, if clinically acceptable, to 0 cm H_2_O. The stability of each steady state was evaluated by a stable end-expiratory EIT signal and a stable arterial saturation. Before the end of each PEEP level, EIT was measured during two minutes and hemodynamic and ventilatory parameters were recorded. Dynamic compliance was calculated by dividing expiratory tidal volume by the constant driving pressure set for each patient. In addition, arterial blood gas analysis was performed (ABL 700, Radiometer, Copenhagen, Denmark) in order to calculate the PaO_2_/FiO_2 _ratio.

**Table 1:** Data on patient characteristics

	Without lung disorders (group N)	With lung disorders (group D)	*P-v*alue
Number of patients	6	8	
Gender, female/male	3/6	3/8	ns
Age (years)	57 (6)	56 (15)	ns
BMI	22.4 (1.8)	24.3 (5.5)	ns
Time of mechanical ventilation (hours)	18.0 (12.5)	8.0 (16.3)	ns
Baseline PEEP (cm H_2_O)	5.0 (0.0)	10.0 (1.0)	0.001
Baseline EELV (L)	1.65 (0.55)	1.2 (1.2)	ns
Baseline PaO_2_/FiO_2 _ratio, (kPa)	59.8 (8.4)	33.5 (6.7)	<0.01
Baseline FiO_2_	35.0 (3.8)	50.0 (10.0)	<0.01
Baseline dynamic compliance (ml/cm H_2_O)	40.1 (12.3)	34.7 (15.6)	ns
Reasons for mechanical ventilation	-Postoperative(n = 4) -Neurological (n = 2)	-Pneumonia (n = 5) -Abdominal sepsis (n = 3)	

Data are presented as median and (where appropriate) interquartile range. BMI, body mass index; PEEP, positive end-expiratory pressure; EELV, end-expiratory lung volume; ns, nonsignificant.

EIT data were stored and analyzed offline on a personal computer (Dell, P4, 2.4 GHz, Round Rock, Texas, USA). The EIT scans consist of images of impedance with a 32 × 32 color-coded matrix relative to the lowest impedance during the PEEP trial (rel. ΔZ). The difference between rel. ΔZ at the end of inspiration and expiration is defined as tidal impedance variation (TIV). This tidal impedance variation is visualized in the functional EIT (fEIT) image, which contains tidal impedance variation per pixel (32 × 32 matrix) averaged over one minute (Figure 1). For analysis of the regional distribution of ventilation, the EIT images were subdivided into two symmetrical non-overlapping ventral to dorsal oriented layers defined as dependent and non-dependent regions of interest (ROI). fEIT images before each PEEP step were subtracted from fEIT images at the end of the next PEEP step to obtain ventilation change maps or ΔfEIT images. This ΔfEIT image represents the change in tidal impedance per pixel. With pressure-controlled ventilation with constant driving pressure, the change in tidal impedance per pixel represents the change in tidal volume per pixel or pixel compliance [7]. A weighted pixel count was used to evaluate total increase and decrease in TIV between PEEP steps in the ΔfEIT images and expressed as the percentage positive of total impedance change by equation 1. In this equation a value of 50% represents a balance between gain and loss of TIV at a given PEEP step.(1)

### Statistical analysis

Statistical analysis was performed with Graphpad software package (version 5.0, Graphpad Software Inc., San Diego, CA, USA). Due to the small number of patients, results are expressed as median and interquartile range. Regional compliance changes between each PEEP step were evaluated with the Wilcoxon matched pair test. EELV, PaO_2_/FiO_2 _ratio and dynamic compliance was evaluated using ANOVA for repeated measurements. The percentage positive of the total tidal impedance change during each PEEP step between both groups was evaluated with the Mann-Whitney U test. For all comparisons, *P *<0.05 was considered significant.

## Results

Table 1 presents data on the 14 mechanically ventilated patients. Group N (n = 6) consisted of postoperative patients (two transhiatal esophagectomy, one liver transplantation and one kidney transplantation) without evidence of pulmonary complications (n = 4), and patients requiring ventilatory support after traumatic brain injury (n = 2). Group D (n = 8) consisted of patients with pneumonia (n = 5) and respiratory failure associated with abdominal sepsis (n = 3). Eight patients were measured at a PEEP of 15, 10 and 5 cm H_2_O and six patients at a PEEP of 15, 10, 5 and 0 cm H_2_O. Respiratory data during the PEEP steps are presented in Table 2. One patient in the group without lung disorders had a transient drop in blood pressure at PEEP 15 cm H_2_O and measurements were continued at 10 PEEP cm H_2_O. All other patients tolerated the PEEP trial well.

**Table 2:** Respiratory parameters during the decremental PEEP steps

PEEP [cm H_2_O]		15	10	5	0	Significance between groups
**EELV (L)**	**N**	2.3 (0.3)	2.2 (0.2)	1.8 (0.2)*	1.5 (0.2)	
	
	**D**	1.9 (1.0)	1.6 (1.0)*	1.3 (0.8)*	1.2 (0.6)	*P *= NS
	
**PaO**_2_**/FiO**_2 _ **(kPa)**	**N**	62.3 (1.9)	63.7 (9.4)*	58.3 (13.9)	50.3 (13.8)	
	
	**D**	37.9 (10.5)	39.0 (8.6)	33.5(15.2)	26.8 (5.1)	*P *<0.01
	
**Cdyn** **(ml/cm H_2_O)**	**N**	38.6 (7.4)	45.0 (4.5)	45.0 (4.6)	41.0 (1.2)	
	
	**D**	34.1 (11.0)	37.0 (12.6)*	35.0 (13.3)*	29.0 (7.1)	*P *= NS

Data are presented as median and (where appropriate) interquartile range. N, without lung disorders, D, with lung disorders. PEEP, positive end-expiratory pressure; EELV, end-expiratory lung volume; Cdyn, dynamic compliance. Asterisk represents significance within groups vs. 15 cm H_2_O.

**Figure 2:**
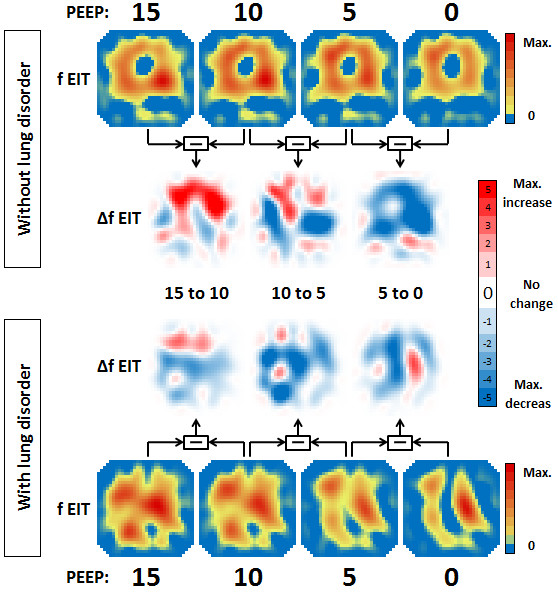
**The effect of a decremental PEEP trial on regional ventilation shown in two representative patients**. The functional EIT (fEIT) image at the different PEEP levels (15 to 10 to 5 to 0 cm H_2_O) shows the ventilation distribution in a colour-coded matrix in a patient without lung disorders and a patient with lung disorders. The ΔfEIT images are created by subtracting fEIT before the PEEP step from fEIT after each PEEP step. The increase or decrease in regional ventilation between PEEP (ΔfEIT) steps is displayed in a color-coded matrix. Each EIT image represents a thoracic slice with the ventral lung regions at the top and dorsal lung regions at the bottom.

Figure 2 exemplarily shows the effect of PEEP on regional ventilation in a patient with lung disorder and in a patient without lung disorder. The ventilation distribution is presented as ventilation distribution maps (fEIT) at each PEEP level and ventilation distribution change maps (ΔfEIT) between PEEP levels. A clear difference can be seen in response to the change in PEEP in the non-dependent and dependent lung regions between these two patients (Figure 2).

**Figure 3:**
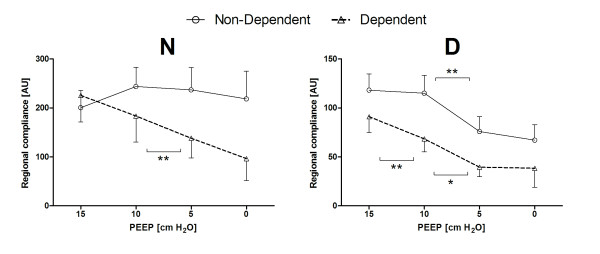
**Changes in regional compliance in patients without (left) and patients with (right) lung disorders**. During pressure-controlled ventilation with constant driving pressure, the tidal impedance change per pixel can be regarded as regional compliance change per pixel. The open triangle represents the dependent lung region and the open circle represents the non-dependent lung region at the different used PEEP levels. Data are presented as mean and SEM. Significance: * *P *<0.05; ** *P *<0.01.

In Figure 3, the tidal impedance per cm H_2_O driving pressure is presented for two ventral to dorsal ROIs at all PEEP levels. In both groups, tidal impedance decreased towards 0 cm H_2_O PEEP. However, this was different for the individual ROIs. In Group N, tidal impedance increased in the non-dependent ROI after decreasing PEEP from 15 to 10 cmH_2_O, whereas decreased in the dependent ROI during each PEEP step. In Group D, tidal impedance variation was significantly lower compared to Group N in both regions. Further, tidal impedance did not change in the non-dependent region between PEEP steps 15 to 10 and 5 to 0 and in the dependent region between PEEP step 5 to 0. Tidal impedance decreased significantly during the other PEEP changes.

**Figure 4:**
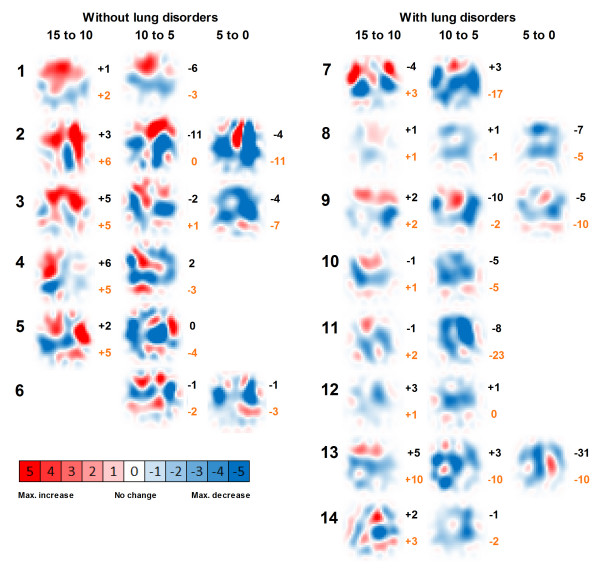
**ΔfEIT images in patients without (1 to 6) and with (8 to 14) lung disorders between the PEEP steps used**. PaO_2_/FiO_2 _ratio change (black) and compliance change (red) are presented next to each ΔfEIT image. Images containing a colour-coded 32 × 32 matrix, are generated by subtracting fEIT before the PEEP step from fEIT after each PEEP step. PEEP is decreased stepwise from 15 to 0 cm H_2_O. Each EIT image represents a thoracic slice with the ventral lung regions at the top and dorsal lung regions at the bottom.

The ΔfEIT images between PEEP steps for the individual patient are shown in Figure 4. In this figure, also the change in PaO_2_/FiO_2 _ratio and change in dynamic compliance during each PEEP step is presented.

**Figure 5:**
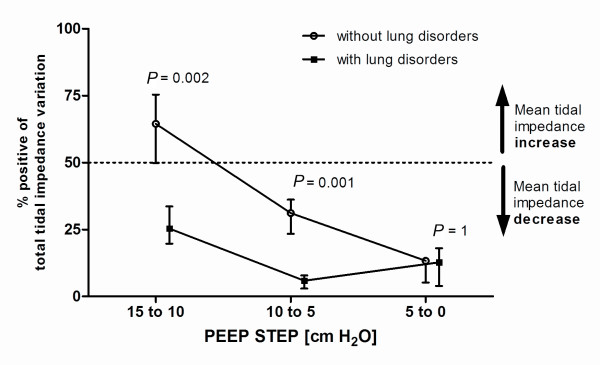
**Response to decremental PEEP steps on tidal impedance change in patients with or without lung disorders**. During pressure-controlled ventilation with constant driving pressure, the tidal impedance change per pixel can be regarded as regional compliance change per pixel. The percentage positive of the total Δtidal impedance is calculated from the total increase and total decrease in each Δfunctional EIT image. Data are presented as median and interquartile range.

In Figure 5 the total impedance change is shown for each PEEP step. Only during the PEEP step from 15 to 10 the increase was higher than the decrease whereas in all other step the decrease was more than the increase. In all patients, the response of regional tidal impedance to PEEP showed a significant difference from 15 to 10 cm H_2_O (*P *= 0.002) and from 10 to 5 cm H_2_O (*P *= 0.001) between patients with and without lung disorders (Figure 5).

## Discussion

This trial shows that EIT is suitable for bedside monitoring of tidal impedance or regional compliance during decremental PEEP steps and can differentiate between dependent and non-dependent lung regions. There was a significant difference in response to a stepwise decrease in PEEP between patients with and without lung disorders, indicating a different PEEP dependency between these two groups.

Using EIT, increase or decrease of tidal impedance variation becomes visible in ΔfEIT maps (Figure 4). During a decremental PEEP trial, as used in the present study, improvement in tidal impedance variation can be caused by recruitment of non-ventilated collapsed alveoli or increased ventilation in previously overdistended alveoli. To obtain an equal balance between derecruitment and overdistension, a value of 50% increase of the total tidal impedance change per ΔfEIT map could be used during decremental PEEP steps. If this value is above 50%, more reduction in overdistention compared to increase in derecruitment is present, whereas below 50%, derecruitment is predominant in the measured EIT slice. In the group without lung disorders, total tidal impedance increased after reducing the PEEP from 15 and from 10 cm H_2_O; this was due to increased tidal impedance variation in the ventral lung regions, with less loss in the dorsal lung regions (Figures 3 and 5). This indicates that during 15 cm H_2_O of PEEP alveoli in the ventral part were inflated throughout the ventilatory cycle or overdistended and after lowering the PEEP to 10 cm H_2_O ventilation increased in the non-dependent part. In this group, after further lowering the PEEP from 10 to 5 cm H_2_O, tidal impedance increase in the ventral part was exceeded by loss of the tidal impedance in the dorsal part; total impedance change was below 50% during this latter PEEP step (Figure 5). In the group with lung disorders, for each PEEP step there was less increase of tidal impedance in the ventral lung regions and more decrease in tidal impedance in the dorsal regions; total positive impedance change was below 50% for each PEEP step (Figure 5). Therefore, in order to prevent alveolar collapse in the dorsal part in this caudal thoracic EIT level, a higher PEEP level should be used in these patients with lung disorders compared to the group without lung disorders (Figures 3 and 5).

EIT is able to show the effect of PEEP on tidal impedance change in each pixel of the functional image matrix. If one accepts the proposed linear relationship between impedance and tidal volume [11-13], the Δimpedance per pixel reflects Δtidal volume in the individual pixel. During pressure-controlled ventilation, the volume change with constant driving pressure or by diving impedance by the driving pressure can be regarded as regional compliance change in each pixel [7].

In the present study, each patient showed an individual response in tidal impedance change during each stepwise decrease in PEEP, and the response differs between dependent and non-dependent lung regions (Figure 4). This is in accordance with the common understanding of gravity-dependent tidal volume distributions in acute lung injury and acute respiratory disease syndrome [14,15]. While the PEEP setting is often guided by global compliance measurements [16-18] or pressure-volume curve analysis [19,20], these global indicators cannot discriminate between the dependent and non-dependent lung regions. EIT is capable of monitoring regional ventilation distribution at the bedside [21-23]. Setting PEEP by the use of tidal impedance or regional compliance requires a definition of the 'optimal' PEEP with regard to the balance between decreased alveolar surface stress and increased alveolar collapse during stepwise decrease in PEEP. In order to minimize ventilator-induced lung injury [24], it is proposed that the lung should be opened by a recruitment maneuver and thereafter a relatively adequate PEEP should be used to keep the lung open, with the lowest inspiratory airway pressure to prevent alveolar overdistention [25]. In the present study, we did not use a recruitment maneuver; however, we have shown that a compromise must be found between lowering alveolar overdistention in the non-dependent part and prevention of alveolar collapse in the dependent part. If optimal PEEP is defined as an equal balance between increase and decrease in tidal impedance, at this thoracic EIT level optimal PEEP would be in the studied patients around 10 cm H_2_O in patients without lung disorders, and at least 15 cm H_2_O in patients with lung disorders (Figure 5). Because EIT is now able to adequately visualize regional change in ventilation, a definition is needed if this method is used to set the optimal PEEP. In the future, three-dimensional EIT could help to monitor the entire lung.

A potential limitation of the present study is that EIT measures an eclipse with a central diameter of 5 to 10 cm. The individual pixel in the EIT matrix contains a vast number of alveoli; during tidal ventilation and/or stepwise change in PEEP, different alveoli might be included in the EIT pixel. However, because the individual pixel contains alveoli from the same lung region, it is unlikely to influence the results of this study. In the present study, we didn't compare our findings to another imaging technique like CT scanning. However, Meier *et al*. used the same method in an experimental animal study and showed good agreement between CT and regional ventilation [9]. In addition, for future studies smaller decremental PEEP steps should be chosen in order to establish the optimum PEEP setting for each individual patient.

## Conclusions

We conclude that during a decremental PEEP trial in ICU patients, EIT measurements performed just above the diaphragm clearly visualize improvement or loss of ventilation in dependent and non-dependent parts, at the bedside in the individual patient. There was a significant difference in response to a stepwise decrease in PEEP between patients with and without lung disorders, indicating a different PEEP dependency between these two groups. However, the individual response to the decrease of PEEP within the groups was also different indicating that optimal PEEP should be titrated individually and can not be generalized for a group of patients. In addition, the response to the decrease of PEEP was different between dependent and non-dependent lung regions within a patient, suggesting that optimal PEEP may be defined as an equal balance between increase and decrease in tidal impedance. This definition of the optimal PEEP in order to minimize VILI needs further research to prove its benefit.

## Key messages

• EIT is suitable for bedside monitoring of tidal impedance or regional compliance during decremental PEEP steps and can differentiate between dependent and non-dependent lung regions.

• During a decremental PEEP trial in ICU patients, EIT measurements performed just above the diaphragm clearly visualize improvement and loss of ventilation in dependent and non-dependent parts, at the bedside in the individual patient.

• Differences in response to decremental PEEP steps were found not only between patient groups, but also within groups indicating that the optimal PEEP should be titrated individually and can not be generalized for a group of patients.

• A definition of the optimal EIT PEEP is needed if this technique is going to be used in the clinical setting.

## Abbreviations

COPD: Chronic Obstructive Pulmonary Disease; CT: computed tomography; EIT: Electrical Impedance Tomography; fEIT: functional EIT; ΔfEIT: functional EIT images subtracted pixelwise from each other; Group D: group with lung disorders; Group N: group without lung disorders; ICU: intensive care unit; PEEP: positive end-expiratory pressure; ROI: region of interest; TIV: tidal impedance variation; ΔZ: mpedance change.

## Competing interests

The authors declare that they have no competing interests.

## Authors' contributions

IB carried out the data acquisition, analysis, statistical analysis and participated in drafting the manuscript. DRM participated in the statistical analysis and in drafting the manuscript. DG participated in the data acquisition and in drafting the manuscript. SL and JB participated in drafting the manuscript. All authors read and approved the final manuscript.

## Acknowledgements

The EIT equipment was supplied by Dräger Medical AG, Lübeck, Germany. The authors thank Laraine Visser-Isles for English language editing.
